# Genetic dissection of growth traits in a Chinese indigenous × commercial broiler chicken cross

**DOI:** 10.1186/1471-2164-14-151

**Published:** 2013-03-06

**Authors:** Zheya Sheng, Mats E Pettersson, Xiaoxiang Hu, Chenglong Luo, Hao Qu, Dingming Shu, Xia Shen, Örjan Carlborg, Ning Li

**Affiliations:** 1 State Key Laboratory for Agro-Biotechnology, China Agricultural University, Beijing, People’s Republic of China; 2 Division of Computational Genetics, Department of Clinical Sciences, Swedish University of Agricultural Sciences (SLU), Uppsala, Sweden; 3 State Key Laboratory of Livestock and Poultry Breeding, Guangzhou, People’s Republic of China

**Keywords:** QTL, Indigenous chicken, Growth, Epistasis, SNP chip

## Abstract

**Background:**

In China, consumers often prefer indigenous broiler chickens over commercial breeds, as they have characteristic meat qualities requested within traditional culinary customs. However, the growth-rate of these indigenous breeds is slower than that of the commercial broilers, which means they have not yet reached their full economic value. Therefore, combining the valuable meat quality of the native chickens with the efficiency of the commercial broilers is of interest. In this study, we generated an F_2_ intercross between the slow growing native broiler breed, Huiyang Beard chicken, and the fast growing commercial broiler breed, High Quality chicken Line A, and used it to map loci explaining the difference in growth rate between these breeds.

**Results:**

A genome scan to identify main-effect loci affecting 24 growth-related traits revealed nine distinct QTL on six chromosomes. Many QTL were pleiotropic and conformed to the correlation patterns observed between phenotypes. Most of the mapped QTL were found in locations where growth QTL have been reported in other populations, although the effects were greater in this population. A genome scan for pairs of interacting loci identified a number of additional QTL in 10 other genomic regions. The epistatic pairs explained 6–8% of the residual phenotypic variance. Seven of the 10 epistatic QTL mapped in regions containing candidate genes in the ubiquitin mediated proteolysis pathway, suggesting the importance of this pathway in the regulation of growth in this chicken population.

**Conclusions:**

The main-effect QTL detected using a standard one-dimensional genome scan accounted for a significant fraction of the observed phenotypic variance in this population. Furthermore, genes in known pathways present interesting candidates for further exploration. This study has thus located several QTL regions as promising candidates for further study, which will increase our understanding of the genetic mechanisms underlying growth-related traits in chickens.

## Background

China has a wide variety of indigenous chicken breeds, most of which can only be found locally in rural areas. Because these breeds are geographically dispersed and have not been subjected to intense artificial breeding, they display unique characteristics as a result of the local environment and/or different breeding objectives than for commercial chicken. Because of the different traditional food cultures across China, native broiler chickens have meat quality characteristics that are often favoured by consumers over those of commercial breeds. Several previous studies found that such characteristics include greater tenderness and preferred flavours [1-3]. Therefore, the native chicken breeds not only contribute to the conservation of poultry genetic resources, but are also of high economic value.

To evaluate the potential for genetic improvement of the productive efficiency of the native Huiyang Beard chicken (HB) breed while maintaining its valuable market properties, including high meat quality and unique appearance, we established an F_2_ intercross between the slow-growing HB and the fast-growing commercial broiler breed “High Quality chicken Line A” (HQLA). We aimed to identify genes contributing to differences in productivity between these populations. Our first objective was to explore the genetic basis of the measured growth-related traits in this F_2_ cross.

In broilers, most economically important traits are growth-related. In this study we focused on six groups of such traits, which we expect to have been under positive selection during the commercial breeding of broiler chickens, including body weight at different ages, and efficiency of feed conversion. QTL mapping has previously been used to determine genomic regions affecting such quantitative traits in other mapping populations, and has identified a large number of QTL [4-7].

As these QTL are generally mapped with low marker density, lack of the genomic information could lead to the less accurately estimated recombination frequency or fail in detecting all the recombination events within the cross. Therefore, additional efforts by utilizing the recently developed genotyping platforms, which can rapidly and economically genotype a high density of SNP markers and have been widely applied to major farm animal species, such as cattle [8], pigs [9] and chickens [10], can be helpful in replicating and confirming these QTL.

In the last few years, the interest in identifying epistatic QTL effects has increased. Epistasis is when the combination of alleles at two, or more, loci yields a phenotype that cannot be explained by the independent effects of the involved loci [11-15]. A number of epistatic QTL have been identified in chicken for growth traits [6,7,16,17], and here we further explored the importance of gene-by-gene interactions in the genetic architecture of growth traits in this intercross.

The aim of the present study was thus to identify the main loci contributing to the phenotypic growth variability in this indigenous × commercial broiler F_2_ intercross, and estimate their direct and epistatic effects by conducting a genome-wide linkage analysis.

## Results

All phenotypic measurements, units, and abbreviations are summarised in Table 1.

**Table 1:** Trait measurements and abbreviations used in this study

**Trait**, **units**	**Abbreviation**
Live body weight at 2 weeks of age, g	BW2
Live body weight at 4 weeks of age, g	BW4
Live body weight at 6 weeks of age, g	BW6
Live body weight at 8 weeks of age, g	BW8
Live body weight at 10 weeks of age, g	BW10
Live body weight at 12 weeks of age, g	BW12
Growth rate at 0–4 weeks of age, g	GR 0–4
Growth rate at 4–8 weeks of age, g	GR 4–8
Growth rate at 8–12 weeks of age, g	GR 8–12
Shank circumference at 4 weeks of age, cm	SC4
Shank circumference at 6 weeks of age, cm	SC6
Shank circumference at 8 weeks of age, cm	SC8
Shank circumference at 10 weeks of age, cm	SC10
Shank circumference at 12 weeks of age, cm	SC12
Shank length at 4 weeks of age, cm	SL4
Shank length at 6 weeks of age, cm	SL6
Shank length at 8 weeks of age, cm	SL8
Shank length at 10 weeks of age, cm	SL10
Shank length at 12 weeks of age, cm	SL12
Stomach weight, g	SW
Abdominal fat weight, g	AFW
Feed conversion ratio at 6–8 weeks of age	FCR 6–8
Feed conversion ratio at 8–10 weeks of age	FCR 8–10
Feed conversion ratio at 10–12 weeks of age	FCR 10–12

### Trait correlations

The Pearson’s correlations between all pairs of phenotypes conformed to expectations, but they varied in strength (Additional file 1: Table S1). There were high and positive correlations (r > 0.5) between live body weight (BW), growth rate (GR), shank length (SL), and shank circumference (SC). Feed conversion ratio (FCR) was negatively correlated (r > −0.5) with stomach weight (SW), BW and GR, and positively correlated with abdominal fat weight (AFW). AFW was negatively correlated (r = −0.36) with SL at the later stages of growth. All the correlations mentioned above were significantly different from zero (p < 0.001).

### One-dimensional QTL scan

**Figure 1:**
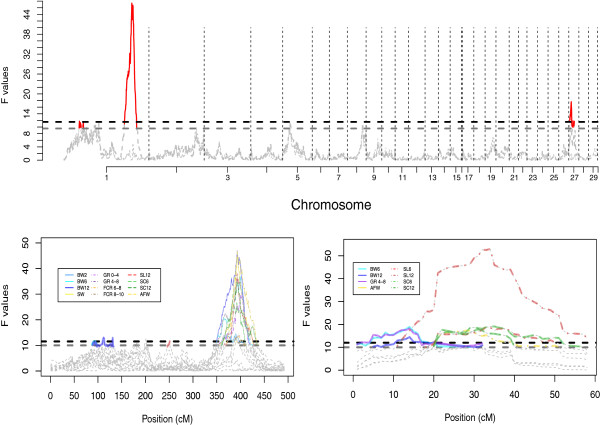
**QTL profiles from the one-dimensional scan of the indigenous × commercial broiler F_2_ intercross. a**) Genome-wide QTL profile for body weight at 10 weeks of age trait (BW10), **b**) and **c**) Chromosome scans for the six groups of phenotypes for GGA1 and GGA27, respectively. In a), the dashed black and grey horizontal lines denote the 1% and 5% genome-wide significance thresholds, respectively, while dashed vertical lines separate the chromosomes. Curves are dashed and grey in non-QTL regions and coloured in red for inferred QTL regions. **b**) and **c**) show the two major QTL on GGA1 and GGA27 detected in our study to illustrate the consistent peaks among different traits. For those traits with coherent records throughout the whole growth period, only the ones representing different growth stages are included.

We performed QTL analyses for 24 traits related to body-size in chickens. Forty-four QTL were detected for 22 traits (no QTL were found for GR 8–12 and FCR 10–12) on six chromosomes at two different significance levels. Five QTL were genome-wide significant at the 5% level, whereas all others were significant at the 1% level (Table 2; Additional file 1: Table S2). Figure 1a shows the genome-wide QTL profile for BW10, while Figure 1b and 1c present the QTL profiles for chromosomes 1 and 27 for several selected traits from all six groups (BW, GR, SC, SL, FCR and Carcass traits).

**Table 2:** QTL affecting growth traits measured in this study

**QTL**	**Trait**	**QTL (cM)**	**Peak marker**	**F value**^**1**^	**Additive effect ± SE**	**Var%**^**2**^
**CAU**_**AB 1a**	BW6	94	rs13849470	11.7**	28.8 ± 5.9	3.9%
	BW8	89	rs15225667	12.3**	42.9 ± 8.7	3.9%
	BW10	89	rs15225667	11.4*	54.6 ± 11.6	3.3%
	BW12	111	rs13858917	13.0**	76.0 ± 15.1	3.8%
**CAU**_**AB 1b**	SL12	250	rs13910430	11.6*	−0.84 ± 0.21	3.0%
**CAU**_**AB 1c**	BW2	389	rs13552715	14.6**	6.3 ± 1.2	5.3%
	BW4	399	rs13974249	30.3**	26.2 ± 3.5	10.8%
	BW6	398	rs15501880	36.9**	53.9 ± 6.4	12.9%
	BW8	398	rs15501880	43.4**	89.0 ± 9.7	14.9%
	BW10	392	rs13972116	47.4**	125.6 ± 13.0	16.0%
	BW12	392	rs13972116	47.0**	154.1 ± 15.9	15.9%
	GR 0–4	399	rs13974249	30.3**	26.1 ± 3.5	10.8%
	GR 4–8	392	rs13972116	15.3**	38.8 ± 7.0	5.6%
	SC6	391	rs14916980	26.7**	0.06 ± 0.01	9.5%
	SC8	391	rs14916980	19.7**	0.06 ± 0.01	7.1%
	SC10	392	rs13972116	30.8**	0.07 ± 0.01	10.9%
	SC12	392	rs13972116	35.1**	0.09 ± 0.01	12.3%
	SL10	392	rs13972116	20.9**	1.4 ± 0.21	6.3%
	SL12	392	rs13972116	38.0**	2.1 ± 0.24	10.9%
	FCR 6–8	396	rs13973293	18.8**	−0.15 ± 0.02	6.8%
	FCR 8–10	389	rs13552715	17.2**	−0.14 ± 0.03	6.2%
	SW	392	rs13972116	44.5**	2.2 ± 0.24	15.2%
	AFW	392	rs13972116	47.0**	−16.1 ± 1.7	16.0%
**CAU**_**AB 2a**	SC4	213	rs1422304	9.8*	−0.03 ± 0.01	3.5%
**CAU**_**AB 2b**	BW2	281	rs13794645	14.0**	5.9 ± 1.1	4.8%
**CAU**_**AB 4**	SC6	152	rs14499051	11.3*	−0.01 ± 0.01	3.4%
**CAU**_**AB 12**	AFW	16	rs14971272	12.3**	−5.2 ± 1.4	3.5%
**CAU**_**AB 27**	BW6	13	rs13620303	18.9**	31.6 ± 6.2	5.4%
	BW8	13	rs13620303	20.5**	46.9 ± 9.3	5.8%
	BW10	13	rs13620303	17.6**	56.0 ± 12.3	5.4%
	BW12	13	rs13620303	14.5**	70.9 ± 15.3	4.4%
	GR 4–8	11	rs14302116	12.4**	30.1 ± 6.6	4.3%
	SC6	34	rs15242584	19.2**	0.05 ± 0.01	6.3%
	SC8	30	rs16047281	19.9**	0.05 ± 0.01	6.7%
	SC10	29	rs16040742	23.9**	0.06 ± 0.01	7.7%
	SC12	30	rs16047281	17.9**	0.06 ± 0.01	5.7%
	SL4	21	rs15241178	13.1**	0.63 ± 0.13	4.7%
	SL6	32	rs14303761	18.9**	0.83 ± 0.14	6.9%
	SL8	32	rs14303761	23.1**	1.1 ± 0.17	8.3%
	SL10	32	rs14303761	49.5**	2.0 ± 0.20	16.6%
	SL12	33	rs16207882	53.0**	2.4 ± 0.23	17.6%
	AFW	27	rs16719300	17.3**	−8.7 ± 1.5	5.3%
**CAU**_**AB 28**	SC10	5	rs16209969	12.0**	−0.03 ± 0.01	3.5%
	SC12	7	rs15246230	11.9*	−0.04 ± 0.01	3.6%

^1^ *5% genome-wide significance. **1% genome-wide significance. ^2^ the percentage of phenotypic variation explained by the detected QTL.

As shown in Table 2, most of the detected QTL clustered to distinct locations in the genome. The exception were the QTL mapped on GGA27, where peaks were distributed across two overlapping clusters - one cluster centred around 13 cM and the other around 30 cM. Fitting both these locations in a two-QTL model did not allow us to discriminate whether there were two independent signals on the chromosome, or if they represented the same QTL with slightly shifted peaks for the different traits. Further analyses in other populations are needed to explore this region further.

The 44 detected QTL were categorized into nine independent QTL. We named each QTL by combining an abbreviation for the cross (China Agricultural University hqlA × hB; *CAU*_*AB*) and the number of the chromosome where the QTL was located. In addition, when multiple distinct QTL were located on the same chromosome, letters were added at the end indicating their order along the chromosome. For example, *CAU*_*AB 1a* represent the most proximal QTL on GGA1.

Four out of the nine independent QTL affected several of the analyzed traits and were therefore classified as being pleiotropic. QTL *CAU*_*AB 1a* affected the intermediate to late growth stage of BW, while *CAU*_*AB 28* was associated with the late growth phase of SC. Two QTL (*CAU*_*AB 1c* and *CAU*_*AB 27*) affected almost all measured growth traits in this cross, which was consistent with the fact that high correlations existed among the traits affected by these QTL. *CAU*_*AB 1c*, on the other hand, affected FCR and the early stage of BW, traits that were not significantly correlated. The remaining five QTL only affected single traits. For the traits recorded at multiple time-points in life, such as BW, SC, and SL, differences were found in the sets of QTL controlling the traits in the different growth stages. More QTL, with larger average effects, were found for growth at intermediate and late growth phases than for early growth.

Positive additive effects, implying enhanced growth associated with the alleles originating from the fast-growing HQLA line, were observed for most of the traits. Most of the exceptions (i.e. QTL with negative additive effects) were detected for AFW and SC. The HQLA line is leaner than HB, and consequently this observation is consistent with the expectations, as a negative additive effect indicates that HB alleles increase AFW. Interestingly, both lines had positive effects on SC in different QTL regions. The proportions of residual phenotypic variance explained by the detected QTL ranged from 3–18%, with the largest effects from *CAU*_*AB 1c* and *CAU*_*AB 27*. It is worth to note that estimation of the genetic variance associated with the detected QTL is generally biased upward [18,19]. In a population of the size used here, only a slight upward bias is expected and therefore the estimates are provided to facilitate a comparison of the relative contribution of the inferred QTL to the observed phenotypic variance in this intercross population.

### Two-dimensional QTL scan

#### Epistatic QTL mapping

We observed significant epistatic interactions for four traits in 10 distinct regions on 10 chromosomes (Table 3; Additional file 1: Table S3). By also examining suggestive interactions (Additional file 1: Table S4), we found that these belonged to coherent peaks, although they did not reach the significance threshold. Therefore, we defined the significant epistatic QTL pairs to be interactions between QTL-peak regions rather than interactions between single marker positions.

**Table 3:** Significant QTL pairs in the two-dimensional epistatic scan in the indigenous × commercial F2 population

**Trait**	**QTL1**^**1**^		**QTL2**^**2**^		**F value**^**3**^	**Var%**^**4**^
	**Chr**^**1**^	**Pos**^**1 **^**(cM)**	**Chr**^**2**^	**Pos**^**2 **^**(cM)**		
**BW6**	4	55	7	100	9.8*	6.8%
**GR 4**–**8**	6	40	25	60	9.5*	6.5%
	1	300	5	120	8.8^+^	6.1%
**FCR 6**–**8**	3	215	26	40	11.1*	8.1%
**FCR 8**–**10**	20	10	22	40	9.4*	6.4%

^1,2^ The first^1^ and second^2^ QTL in the significant epistatic QTL pair and their chromosomal location in centimorgans (cM); ^3^ *5% genome-wide significance; +10% suggestive genome-wide significance.^4^ The percentage of the residual phenotypic variance explained by the epistatic QTL pair.

As shown in Additional file 1: Table S3, additive-by-additive interactions contributed a large proportion of the total epistatic effects for four out of the five interacting pairs. The only exception was for the pair affecting BW6, where the dominance-by-dominance effect was most significant.

(Additional file 2: Figure S2) shows the genotype-phenotype map for the interacting pairs. Each pair had a unique epistatic pattern, indicating complicated interactions between the loci.

In summary, interactions were often identified in the same regions for consecutive growth phases of the BW traits, even though not all reached the significance threshold (Additional file 1: Table S4). This indicates that the identified interacting effects act throughout extended growth periods. The inferred epistatic pairs explained 6–8% of the residual phenotypic variance, which is substantial given the size of the QTL detected in the single-QTL scan.

### Identifying candidate epistatic genes in known biological pathways

**Figure 2:**
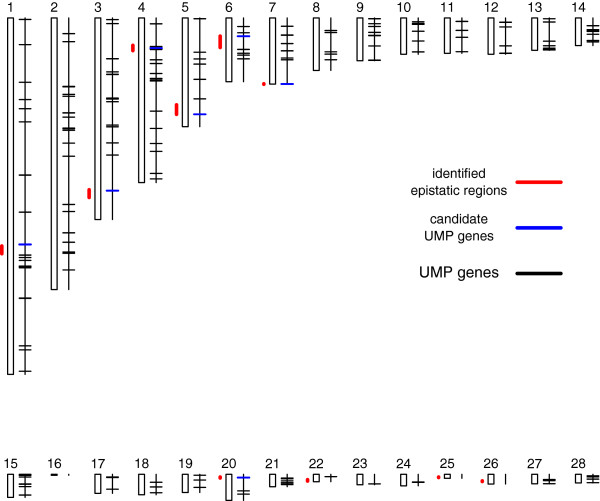
**Localisation of epistatic QTL and corresponding candidate genes from the UMP pathway throughout the genome.** The 28 vertical boxes represent chicken autosomes GGA1–GGA28. All 10 identified epistatic regions are presented as red vertical bars, and UMP genes within the epistatic regions are presented as blue horizontal lines, while other genes are displayed in black.

In contrast to the results from the single-QTL scan, where correlated traits often shared the same QTL, the epistatic analysis identified mostly novel regions (Table 3). When examining the 10 epistatic regions in more detail [20], genes from the same KEGG biological pathway [21], ubiquitin mediated proteolysis (UMP), were found in seven of the 10 regions (Table 4). The genes belonging to the UMP pathway across the genome and in the identified epistatic regions are presented in Figure 2. Using a 10,000-fold permutation test, we estimated that the probability of observing such a seven-locus overlap with the gene from UMP pathway was only 1.6% (Additional file 3: Figure S3). The UMP pathway is a temporally controlled and tightly regulated process, which plays major roles in a variety of basic pathways during cell life and death. Hence, it is crucial for cell growth and differentiation [22]. For the QTL pairs affecting FCR 6–8 and FCR 8–10, we also found possible interacting candidate genes in a growth-related pathway, the ErbB signalling pathway, which is known to affect intracellular signalling pathways regulating cell proliferation, differentiation, cell motility, and survival [23]. Genes in the ErbB pathway were also identified in the epistatic regions for GR 4–8.

**Table 4:** Candidate genes in the epistatic QTL regions and their involvement in growth-related pathways

**Trait**	**Gene**	**Chr**	**Position bp/(cM)**^**1**^	**Pathway**^**2**^
**BW6**	**UBE2A** ubiquitin-conjugating enzyme E2A	4	16506437 – 16512992 (55–55.1)	UMP
	**MARCH7** membrane-associated ring finger (C3HC4) 7	7	36187561 – 36207776 (99.8)	UMP
**GR 4**–**8**	**HERC4** hect domain and RLD 4	6	10024961 – 10054292 (36.7)	UMP
	**ZBTB1** zinc finger and BTB domain containing 1	5	52791938 – 52805593 (135.1)	UMP
	**MID1** midline 1	1	124042084-124134384 (296.9)	UMP
**FCR 6**–**8**	**RNF144A** ring finger protein 144A	3	94598486 – 94657682 (206.8)	UMP
	**NRAS** neuroblastoma RAS viral (v-ras) oncogene homolog	26	3847830 – 3854232 (43.6)	ER
**FCR 8**–**10**	**ITCH** itchy E3 ubiquitin protein ligase homolog	20	1769346 – 1829955 (7.5–7.7)	UMP
	**TGFA** Transforming growth factor alpha	22	3012283 – 3020403 (41.1)	ER

^1^ The physical locations of the genes based on the WASHUC 3.1 build, and the genetic locations of the scored SNP markers located within the gene. ^2^ UMP: Ubiquitin-mediated proteolysis; ER: ErbB signalling.

## Discussion

This study describes the genetic dissection of 22 growth related traits in a chicken F_2_ population. Forty-four QTL were identified, 39 of which belonged to one of four major pleiotropic QTL regions, while the remaining five each affected a single trait. The co-localized QTL did, however, explain different amounts of residual phenotypic variance for different traits. Given the correlations among traits, this high degree of pleiotropy observed for the QTL was not surprising. For instance, the HQLA allele at *CAU*_*AB 1c* increases both growth rate and size of the digestive organs, a correlated response that has been observed in previous physiological studies [24,25]. The pleiotropic effects of this QTL on many traits investigated in this study are in the direction preferred in selection programs for increased productivity, as the HQLA allele improves the performance of traits like BW and SL, while at the same time decreasing AFW and FCR. These characteristics of the QTL make it an interesting candidate for further studies to better understand the molecular mechanisms underlying the response to selection for faster growth in chickens.

The QTL *CAU*_*AB 1c*, which affects many traits in this cross, is located at the distal end of GGA1 (173.7 Mb). This location overlaps with several earlier reported growth QTL in the Chicken QTL Database [26]. Interestingly, it also maps very near to a 1.5-Mb region (173.5–175 Mb) on GGA1, identified in a recent GWAS study examining growth differences between the White Recessive Rock and the Xinghua chicken (another Chinese indigenous breed) [27]. Most of the other QTL mapped in this study also overlap with earlier reported QTL [26]. We did, however, find that these loci have larger genetic effects and higher statistical significance in this study. This could be due to the more informative genetic markers used, but also a reflection of the larger divergence between the founder lines for the studied traits. This would, in turn, indicate that these loci have been important for the selection response while breeding broiler chickens and that there exist multiple, distinct alleles at these loci in the global chicken population. Further in-depth molecular dissection of these loci could therefore help to identify the genetic mechanisms underlying important traits in broiler breeding.

No significant result was found for GR 8–12, despite the strong signals detected for both BW8 and BW12. Also, no significant QTL was detected for FCR 10–12, which was consistent with the observation that GR 8–12 was strongly correlated with FCR 10–12. This result indicates that late GR is not controlled by the same loci as early GR, and that fewer loci with large individual effects appear to be involved in late GR, which is consistent with earlier findings in chicken [16].

The epistatic analysis identified significant interactions for four traits, all affecting the birds between 6–10 weeks of age. This is the same time-period where the highest statistical significance for QTL was found also in the single locus analysis. The epistatic QTL identified at different times appeared to have a much more complicated pattern than that revealed in the single-QTL scan. Even so, for some traits such as BW, we could conclude that the same epistatic QTL pairs were identified for late growth (from 6–10 weeks of age; Additional file 1: Table S4), while different QTL pairs were found for early growth (from 2–4 weeks of age, data not shown). Both the results from the epistatic analysis and the single locus scan are consistent with earlier findings that different sets of genes regulate early and late growth [16,28-30].

(Additional file 2: Figure S2) shows the two-locus genotype-phenotype maps for the detected epistatic pairs. All displayed complicated interaction patterns, two of which were found to be more intriguing. These two pairs affected BW6 and GR84 (Additional file 2: Figure S2a and S2c) and had significantly larger phenotype values when both the interacting QTL were homozygous for the allele with a HQLA origin. This pattern is consistent with the selective breeding scheme that has been applied in the founder lines. Also, it suggests that selection for growth is not acting only on independent alleles at single loci, but also on combinations of alleles at multiple loci.

Compared to the single-QTL analysis, the relatively large genetic variances explained by the pairs detected in the epistatic analysis suggested that epistasis is an important contributor as some of the pairs explained more than the minor single QTL effects. Interestingly, the five epistatic QTL pairs involved 10 independent regions across 10 autosomes, indicating that the detected epistatic loci each provide novel insights into the genetic basis of the studied growth traits. It is somewhat surprising that none of the highly significant QTL detected in one-dimensional analysis were involved in significant epistatic interaction with other regions across the genome. This low degree of overlap between QTL identified in one- and two-dimensional scans, however, illustrates the need to perform both single-QTL and epistatic QTL analysis to better dissect complex quantitative traits.

Most of the epistatic regions contained genes from the UMP, or related, pathways. The UMP pathway contains 204 genes across the chicken genome (Additional file 1: Table S5) [20,21]. A permutation test estimated that the probability of obtaining such an overlap by chance was about 1.6% (Additional file 3: Figure S3), indicating that this result is unlikely to be a coincidence. Further studies of this pathway will hopefully be able to provide new insights to the genetics of growth-related phenotypes.

Because of its cost efficiency and ease of use, we genotyped the birds using a dense SNP chip. While the high marker density does not immediately help to narrow QTL regions because of the lack of recombination events in the F2 design, it does help to increase the precision of the line origin estimation. Much work remains to identify the causal mutations underlying the traits, but two regions are of particular interest. Further studies of the *CAU*_*AB 1c* region on GGA1 and the entire GGA27 are highly promising, as the effects at those loci are particularly strong. In addition, the epistatic analysis identified interesting QTL regions where a set of possible candidate genes that are part of the same biological pathway are located. As sequencing technology becomes more affordable, re-sequencing can be used to search for potential functional polymorphisms between the lines in the highlighted regions as a way to identify the causal genes.

## Conclusion

In summary, we performed a genome-wide QTL analysis in an indigenous × commercial broiler F_2_ intercross. A single-QTL analysis revealed nine distinct QTL regions with significant effects on 22 traits. These QTL were often pleiotropic, which is consistent with the observed correlations between the studied traits, and mostly mapped to regions identified in earlier chicken studies. The genetic effects of these loci were, however, greater in this study than in previous reports. Second, an exhaustive search for epistatic effects identified five distinct interacting QTL pairs. The epistatic QTL did not, however, overlap with the results of the single-QTL analysis, which indicates that epistatic combinations of loci might have contributed to selection response in addition to the single QTL, but that these are more dependent on the allelic combinations available in the founders. For seven of the 10 epistatic QTL, we found candidate genes belonging to the UMP pathway, suggesting this as a potentially important pathway in the regulation of growth in chicken.

## Methods

### Ethics statement

All animals used in the current study were cared for and used according to the requirements of the Institute of Animal Science, Guangdong Academy of Agricultural Sciences (No. GAAS-IAS-2009-73).

### Experimental population

A three-generation intercross population was constructed from two divergent chicken lines. One of the founder lines was the “High Quality chicken Line A” (HQLA), bred by Guangdong Wiz Agricultural Science & Technology Co. (Guangzhou, China), a closed population founded by the commercial Anak chicken breed and an indigenous chicken line unrelated to the other founder line of this study. This population has been under selection for fast growth for more than 10 generations, while maintaining the meat quality. The other founder line was the Huiyang Beard chicken (HB), a native Chinese meat-type breed, which is characterized by slow growth and high meat quality. The average phenotypes, i.e. body weight at consecutive growth phases, are given for each sex in each line in (Additional file 1: Table S6).

The F_2_ cross was generated by reciprocal crossing of the founder lines (details presented in Additional file 4: Figure S4). We denote the cross between a cock from line HQLA and a hen from line HB as cross type A × B, the reverse as cross type B × A. First, four cocks and 12 hens from each line were chosen as the F_0_ generation. The males were full siblings, while the females were either half or full siblings. It is important to note that the cocks and hens of the same line did not share either of their direct parents, thus not closely related. Each cock was mated to three hens and 399 F_1_ individuals were produced. To balance the offspring of the 8 F_0_ cocks in the next generation, 56 chickens from the F_1_ population, all with good health condition and similar body weight, were selected for further crossing. Each cock from cross type A × B was mated with six hens from cross type B × A and vice versa. Forty-eight full-sib families including 800 F_2_ individuals were produced and hatched in six batches.

During the first 5 weeks, the chickens were divided into groups by hatch. Each group was kept in a single cage. A starter diet (2,900 kcal of ME/kg and 200 g/kg of CP) was provided during this period. Then, from weeks 6–13, each individual was reared separately and fed a grower diet (2,950 kcal of ME/kg and 180 g/kg of CP). All chickens had free access to feed and water. The breeding facility supplied 24-hour lighting and was equipped with a water curtain system to control the ambient temperature.

### Phenotyping

BW was measured at hatching and every other week until 12 weeks of age. Using these observations, we calculated three estimates of GR as the weight gain during the periods, which were 0–4, 4–8, and 8–12 weeks of age. During weeks 4–12, SL and SC were also measured every 2 weeks. Anatomical traits such as SW (the combined weight of the ventriculus and the proventriculus) and AFW were recorded at the 13th week, after the birds were slaughtered. FCR was calculated as the ratio between feed intake and body weight gain over the weeks specified.

Boxplots for each phenotype were generated in R [31] to scan for outliers. Individuals that were further than 1.5 times the interquartile range away from the lower or upper quartile of the boxplots were marked for further examination. The majority of the marked data were considered as outliers, and were eliminated from further analysis (data not shown). However, the marked data were not excluded if there were multiple marked data points that were clustered, or if the data points were observed consistently throughout the growth phase. After this procedure, descriptive statistics of the phenotypes in the population were calculated and are provided in Table 5.

**Table 5:** Descriptive statistics for the studied phenotypes

**Traits**	**No**. **of records**	**Mean**	**SD**	**Minimum**	**Maximum**
*BW2* (*g*)	493	167.2	21.6	115.0	251.0
*BW4* (*g*)	491	443.5	74.2	273.0	708.5
*BW6* (*g*)	492	803.1	131.6	439.0	1290.5
*BW8* (*g*)	492	1240.1	208.5	475.5	2005.5
*BW10* (*g*)	493	1662.0	287.2	727.5	2719.5
*BW12* (*g*)	492	2032.2	352.4	1042.0	3250.0
*GR 0*–*4* (*g*)	490	415.9	73.5	246.4	676.8
*GR 4*–*8* (*g*)	490	798.3	154.5	110.5	1297.0
*GR 8*–*12* (*g*)	491	792.5	177.7	20.5	1277.5
*SC4* (*cm*)	493	2.9	0.25	2.2	3.5
*SC6* (*cm*)	493	3.4	0.27	2.7	4.1
*SC8* (*cm*)	493	3.8	0.31	2.9	4.5
*SC10* (*cm*)	493	4.0	0.32	3.3	4.7
*SC12* (*cm*)	493	4.2	0.36	3.1	5.0
*SL4* (*mm*)	493	54.9	3.9	43.0	63.5
*SL6* (*mm*)	493	68.2	4.7	55.5	80.7
*SL8* (*mm*)	493	80.4	6.2	61.6	97.2
*SL10* (*mm*)	493	89.3	8.2	68.1	106.4
*SL12* (*mm*)	493	92.8	10.2	73.5	114.3
*SW* (*g*)	493	19.1	3.9	10.2	36.3
*AFW* (*g*)	490	83.3	33.7	2.3	169.1
*FCR 6*–*8*	490	2.9	0.39	1.9	7.1
*FCR 8*–*10*	492	3.5	0.46	2.1	5.6
*FCR 10*–*12*	486	4.4	0.76	2.9	11.1

### Genotyping and map construction

In total, 585 individuals were genotyped, comprising 22 F_0_ individuals, 52 F_1_ animals, and 511 F_2_ progeny (from 43 of the 48 full-sib families). Families were selected for genotyping were those with the highest quality of the pedigree information and phenotype records. Genomic DNA extraction from blood was performed using the phenol-chloroform method. Genotyping was performed using an Illumina Chicken 60K SNP Beadchip [10] and was performed by DNA LandMarks (Saint-Jean-sur-Richelieu, Canada).

Quality control was assessed using custom Perl [http://www.perl.org/] scripts (available from the authors on request). The QC procedure excluded 24 individuals and 16352 SNPs from the analysis for failing to fulfil one or more of the following criteria: call rate of individuals >0.9, call frequency of SNPs >0.9, minor allele frequency >0.05, and inheritance error rate for either individuals or SNPs <0.05 (The complete genotype data are available in Additional file 5).

An improved version of CRI-MAP [32] was used to construct and validate the genetic map. The result was a sex-average linkage map for 29 autosomal linkage groups spanning about 3068 cM, which is consistent with reported linkage maps in chickens [33]. Further details regarding the linkage map are presented in Table 6.

**Table 6:** Summary statistics for the linkage map and number of informative markers in the F2 population

**Chromosome**	**Physical size**^**1 **^**(Mb)**	**Number of SNPs**	**Sex average (cM)**^**2**^	**Recombination rate (cM/Mb)**^**2**^
**GGA1**	201	6800	491.2	2.4
**GGA2**	155	5073	318.8	2.1
**GGA3**	114	3924	269.3	2.4
**GGA4**	94	3143	183	2
**GGA5**	62	2112	170.1	2.7
**GGA6**	37	1595	98.8	2.7
**GGA7**	38	1627	103.9	2.7
**GGA8**	31	1330	107.4	3.5
**GGA9**	26	1115	90.2	3.5
**GGA10**	22.6	1247	77	3.4
**GGA11**	21.9	1172	77.2	3.5
**GGA12**	20.5	1252	94.1	4.6
**GGA13**	18.9	1090	76.8	4.1
**GGA14**	15.8	968	67.1	4.3
**GGA15**	13	993	65.9	5.1
**GGA16**	0.43	13	0.8	n.d. ^**3**^
**GGA17**	11.2	830	66.8	6
**GGA18**	10.9	826	65	6
**GGA19**	9.9	779	62.8	6.3
**GGA20**	14	1346	75.2	5.4
**GGA21**	7	730	54.2	7.7
**GGA22**	3.9	285	47.4	12.2
**GGA23**	6	562	60.2	10
**GGA24**	6.4	659	60.6	9.5
**GGA25**	2.03	164	61.8	n.d. ^**3**^
**GGA26**	5.1	601	57.5	11.3
**GGA27**	4.8	436	57.8	12.1
**GGA28**	4.5	503	55.5	12.3
**LGE22**	0.9	103	52	n.d. ^**2**^
**Total autosomal**	957.8	41278	3068.4	3.2

^1^ Physical length of the chromosome was based on the WASHUC 3.1 build. ^2^ Estimated from the genetic map estimated in this population and the physical map from the NCBI database. ^3^ n.d. = not determined, as the chromosome showed clear evidence of sequence gaps.

### Statistical analysis

Preliminary models (as shown below) were fit to determine which non-genetic effects should be included in the further analysis of each trait.

(1) y=μ+βF+γC+ε

Here, *y* is the phenotypic value, *μ* is the mean of estimated phenotypic values, *β* is the estimate for fixed effects, *γ* is the estimated effects of covariates, *F* and *C* are the indicator variables of fixed effects and covariates, respectively, and *ε* is a normally distributed residual error.

Although fixed effects, including sex, batch, and family effects, as well as the possible covariates, were considered, only those that had significant effects were included in further analyses (details are available in Additional file 1: Table S2).

### One-dimensional QTL scan

Single QTL were mapped using the least squares regression method as described by Haley et al. (1994) [34]. Based on the genetic map of our population, the probabilities for the line origin combinations, that is, the probabilities of both alleles being inherited from line HQLA (genotype AA), both being inherited from line HB (genotype BB), and one allele being inherited from each line (genotype AB and BA), were calculated using the triM [35] algorithm at every centiMorgan (cM) throughout the genome for all F_2_ individuals. Once the line origin probabilities had been calculated, the coefficients for *a* and *d* for a putative QTL at each cM can be determined as *A* = Pr(*genotypeAA*) – Pr(*genotypeBB*) and *D* = Pr(*genotypeAB*) + Pr(*genotypeBA*), where Pr(*genotypeX*) is the probability of having genotype X. Then, a multiple linear regression model was used to estimate the genetic effects for putative QTL at 1 cM intervals across the genome.

(2) y=μ+βF+γC+aA+dD+ε

where *y*, *μ*, *β*, *γ*, *F*, and *C* are the same as stated in model (1), *a* and *d* are the additive and dominance effects, respectively, of the tested position, and *ε* is a normally distributed residual error. F values were calculated based on model (1) and model (2).

Additive and dominance regression indicator variables of the most significant QTL in each round of analysis were added to the statistical model (2), and another genome scan was performed until no more significant QTL were detected for the analysed trait.

To potentially separate effects of QTL on the same chromosome, we performed a second statistical analysis including both QTLs on the same chromosome in the model. If both QTL effects remained significant after this procedure, we defined them as independent QTL.

### Two-dimensional scan

To explore the interacting effects (*i*, as shown below in model (4)) between two loci, we further used the line origin probabilities to calculate the indicators of *i* by multiplying the indicator variables from locus 1 with those from locus 2 (indicated in the subscripts). For instance, the coefficent for the additive-by-additive effect between two loci can be generated by the formula: *I*_*A*1*A*2_ = *A*_*1*_ × *A*_*2*_ = [Pr(*genotypeAA for locus 1*) – Pr(*genotypeBB for locus 1*)] × [Pr(*genotypeAA for locus 2*) – Pr(*genotypeBB for locus 2*)], where Pr(*genotypeX for locus K*) is the probability of locus K having genotype X.

On the basis of the two-locus models, including the null model (model (3)) and the full model (model (4)), an exhaustive two-dimensional genome scan was performed to detect pairs of epistatic QTL:

(3) $$\begin{array}{l} y=\mu +\mathrm{\beta F}+\mathrm{\gamma C}+a_{1}A_{1}+d_{1}D_{1}+a_{2}A_{2}\\ \;+d_{2}D_{2}+\varepsilon \end{array}$$

(4) $$\begin{array}{l} y=\mu +\mathrm{\beta F}+\mathrm{\gamma C}+a_{1}A_{1}+d_{1}D_{1}+a_{2}A_{2}\\ \;+d_{2}D_{2}+i_{1}I_{A}{}_{1A2}+i_{2}I_{A}{}_{1D2}+i_{3}I_{D}{}_{1A2}\\ \;+i_{4}I_{D}{}_{1D2}+\varepsilon \end{array}$$

where *y*, *μ*, *β*, *γ*, *F*, and *C* are as stated in model (1), *A*_*1*_, *D*_*1*_, *A*_*2*_ and *D*_*2*_ are the additive and dominance indicator variables of the first and second tested loci, *I* values are the indicators of interacting effects, *a*_*1*_, *d*_*1*_, *a*_*2*_ and *d*_*2*_ are the additive and dominance effect, respectively, of the first and second locus (indicated in the subscripts), and *i*_*1*_, *i*_*2*_, *i*_*3*_ and *i*_*4*_ are the additive-by-additive, additive-by-dominance, dominance-by-additive, and dominance-by-dominance interaction effects between two positions. F values were calculated based on models (3) and (4).

The genotype-phenotype maps for detected epistatic pairs were visualised using a discretised estimate of the line origin. Here, only individuals for which the probability of one of the genotypes was higher than 0.8 were included. Then if, for example, Pr(*genotypeAA*) for an individual at a marker was greater than 0.8, it would be assigned the discretised genotype *AA* at this marker. This set of individuals with high-confidence, discretised genotypes in the interacting regions was then used to estimate the residual (ỹ from model (1)) phenotypic means for each two-locus genotype.

The permutation test for the distribution of the number of regions containing genes from the UMP pathway was performed in three steps. First, we allocated each region to a chromosome by sampling with replacement in the range of 1–28, since the epistatic QTL scan was performed only on the 28 autosomes. Second, to best mimic our results, we selected the size of each region by sampling without replacement from the vector of detected regions sizes, found in Additional file 1: Table S3. Each size selection was followed by a validation step, to ensure that the chromosome in question was larger than the sum of the regions placed upon it. Then, the starting-point position of each region was assigned to a random base of the assigned chromosome, and then the stop position was calculated based on the size of that region, with checks in place to make sure the regions were non-overlapping and within the chromosome boundaries. Finally, the number of regions containing UMP genes was scored. This procedure was repeated 10000 times. The final distribution obtained in the test was shown as a histogram (Additional file 3: Figure S3).

The genome-wide significance threshold was determined using a randomisation test based on 1,000 permuted datasets as described by Churchill and Doerge [36]. Because of the computational demand of the randomisation testing, tests for epistasis were performed with 5 cM spacing.

The proportion of the residual phenotypic variance that was explained by the detected QTL was calculated by the following equation:

(5) $$\mathrm{Var\%}=\left(\mathrm{MS}{’}_{R}–MS_{F}\right)/MS_{R}$$

Here, *MS*’_*R*_ is the residual mean square of the reduced model (i.e. model (1) also including other QTL as cofactors or model (3), accordingly), *MS*_*F*_ is the residual mean square of the full model (i.e. model (2) or (4), accordingly), and *MS*_*R*_ is the residual mean square of the reduced model (only for model (1), it fit all covariates, but not any QTL for single-QTL analysis).

All of the statistical analyses were performed in the R statistical framework [31].

## Abbreviations

AFW: Abdominal fat weight; BW: Live body weight; CAU_AB: Abbreviation for the China Agricultural University F2 intercross between line HQLA and line HB; cM: Centimorgan; CP: Crude protein; ER: ErbB signaling; FCR: Feed conversion ratio; GGA: Gallus gallus autosome; GR: Growth rate; HB: Huiyang Beard Chicken; HQLA: High Quality chicken Line A; ME: Metabolic energy; QTL: Quantitative trait locus/loci; SC: Shank circumference; SNP: Single nucleotide polymorphism; SL: Shank length; SW: Stomach weight; UMP: Ubiquitin mediated proteolysis.

## Competing interests

The authors declare that they have no competing interests.

## Authors’ contributions

NL, ÖC, and XH conceived the project, designed and organized the study, CL, HQ, and DS established the experimental population and collected the data, ZS performaned the experiments and conducted the analysis, XS, ÖC and MEP assisted in the data analysis, ZS and MEP drafted the manuscript, ÖC edited the manuscript. All authors read and approved the final manuscript.

## Supplementary Material

Additional file 1: Table S1 Pearson correlations between all growth-related traits used in the analysis. **Table S2.** Complete growth QTL results for all chromosomes in this study. **Table S3.** Complete epistatic QTL results for all detected significant pairs in this study. **Table S4.** Results of a two-dimensional scan including the suggestive interactions (F > 8) detected in the scan. **Table S5.** Full list of UMP-related genes in the chicken genome. **Table S6.** Phenotypic averages for the body weight traits determined at different growth stages for the two divergent lines used as founders for the F_2_ intercross. Click here for file

Additional file 2: Figure S2 Two-locus genotype-phenotype maps for significant epistatic QTL pairs. Genotype-phenotype maps for the five significant two-locus interactions affecting: a) BW6, b) GR 4–8 (*pair of regions in GGA6 and GGA25*), c) GR 4–8 (*pair of regions in GGA1 and GGA5*), d) FCR 6–8, e) FCR 8–10. The letters A and B in the genotypes represent the line-of-origin of alleles from the founder lines HQLA and HB, respectively. Click here for file

Additional file 3: Figure S3 Numerical distribution of the regions containing genes from the UMP pathway from a 10,000-time permutation test. The x-axis is the discrete number of regions (0–10) that contain genes belonging to the ubiquitin-mediated pathway. The y-axis shows the frequency of the corresponding region number observed in the test. The numbers above the rectangles are the actual counts of the corresponding observation from the10,000-time permutation test. Click here for file

Additional file 4: Figure S4 Structure of the reciprocal cross between High Quality chicken Line A (HQLA) and Huiyang Beard chicken (HB) for QTL mapping. Using the last letter from the abbreviation of each line, we described the progeny between cocks from HQLA and hens from HB as “A × B” and vice versa. F_0_, F_1_ and F_2_ animals are labelled in orange, red and purple, respectively. Males are labelled with squares/rectangles, and females are labelled with circles/ovals. Descriptions of animals used in the cross are given in parentheses. The mating between F1 individuals are indicated with blue lines, and labelled with a serial number from 0–8 in dashed circles. In summary, we mated four HQLA cocks with 12 HB hens, and four HB cocks with 12 HQLA hens, yielding 399 F_1_ offspring. To balance the progeny of the eight F_0_ cocks in the next generation, eight F_1_ cocks (four A × B and four B × A) and 48 F_1_ hens (28 A × B and 28 B × A) were chosen for further crossing. Then, each cock from A × B was mated with six hens from B × A and vice versa. Click here for file

Additional file 5 **A txt file containing SNP chip data used in this study.** Columns show the genotype data of each individual, while rows indicate the genotype data of the markers. Click here for file
