# Supplementary figures and images for: Genetic dissection of growth traits in a Chinese indigenous × commercial broiler chicken cross
Source: BMC Genomics. 2013 Mar 6;14:151. doi: 10.1186/1471-2164-14-151 (PMC3679733; doi:10.1186/1471-2164-14-151)

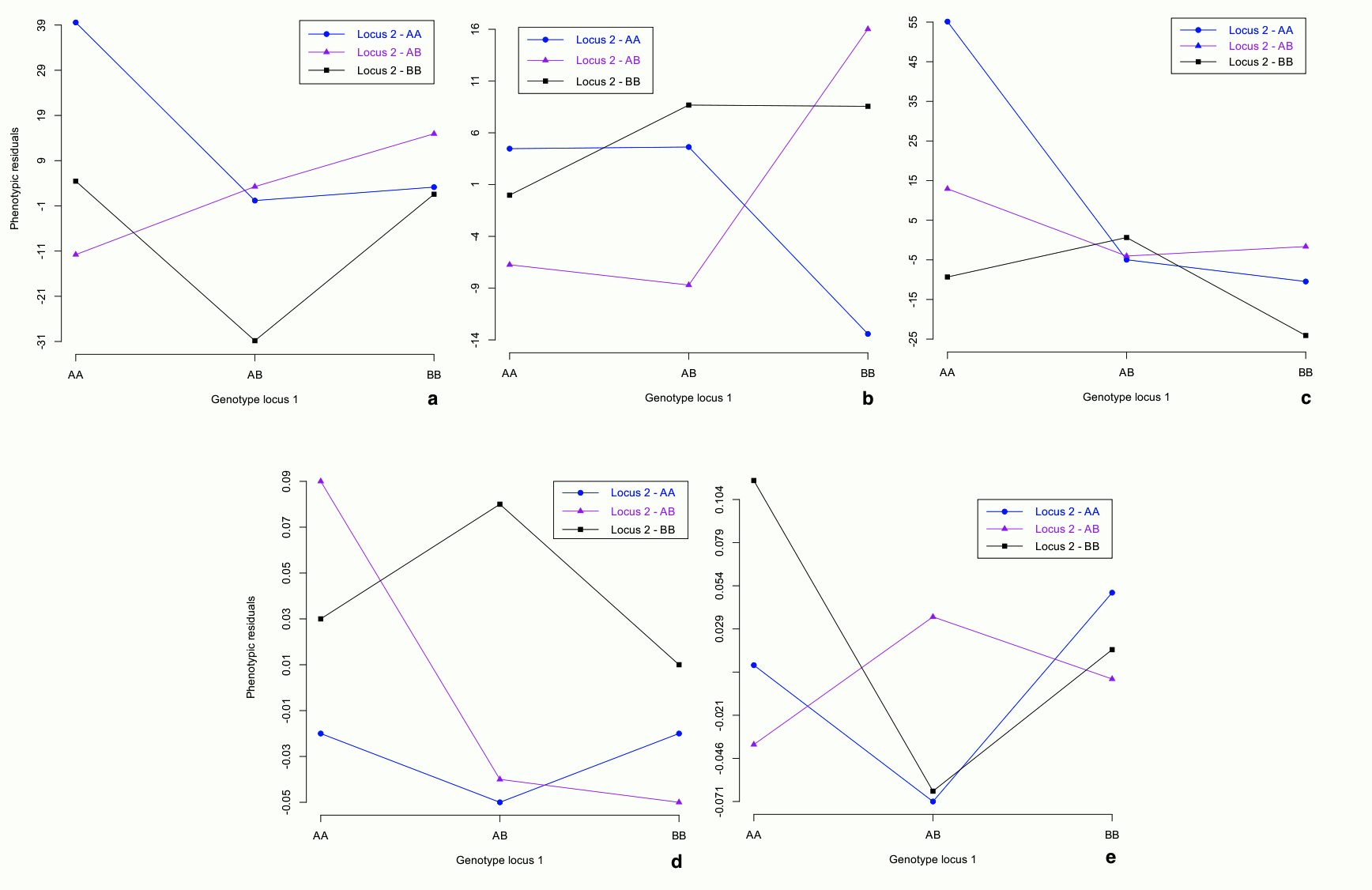

Supplement: Additional file 2: Figure S2 — Two-locus genotype-phenotype maps for significant epistatic QTL pairs. Genotype-phenotype maps for the five significant two-locus interactions affecting: a) BW6, b) GR 4–8 (pair of regions in GGA6 and GGA25), c) GR 4–8 (pair of regions in GGA1 and GGA5), d) FCR 6–8, e) FCR 8–10. The letters A and B in the genotypes represent the line-of-origin of alleles from the founder lines HQLA and HB, respectively. [file 1471-2164-14-151-S2.gif]

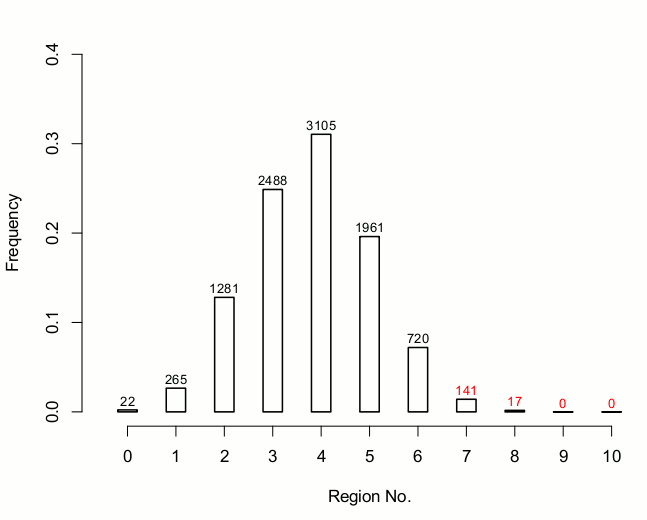

Supplement: Additional file 3: Figure S3 — Numerical distribution of the regions containing genes from the UMP pathway from a 10,000-time permutation test. The x-axis is the discrete number of regions (0–10) that contain genes belonging to the ubiquitin-mediated pathway. The y-axis shows the frequency of the corresponding region number observed in the test. The numbers above the rectangles are the actual counts of the corresponding observation from the10,000-time permutation test. [file 1471-2164-14-151-S3.gif]
