# Supplementary material for: Genetic dissection of growth traits in a Chinese indigenous × commercial broiler chicken cross
Source: BMC Genomics. 2013 Mar 6;14:151. doi: 10.1186/1471-2164-14-151 (PMC3679733; doi:10.1186/1471-2164-14-151)
Supplement: Additional file 4: Figure S4 — Structure of the reciprocal cross between High Quality chicken Line A (HQLA) and Huiyang Beard chicken (HB) for QTL mapping. Using the last letter from the abbreviation of each line, we described the progeny between cocks from HQLA and hens from HB as “A × B” and vice versa. F0, F1 and F2 animals are labelled in orange, red and purple, respectively. Males are labelled with squares/rectangles, and females are labelled with circles/ovals. Descriptions of animals used in the cross are given in parentheses. The mating between F1 individuals are indicated with blue lines, and labelled with a serial number from 0–8 in dashed circles. In summary, we mated four HQLA cocks with 12 HB hens, and four HB cocks with 12 HQLA hens, yielding 399 F1 offspring. To balance the progeny of the eight F0 cocks in the next generation, eight F1 cocks (four A × B and four B × A) and 48 F1 hens (28 A × B and 28 B × A) were chosen for further crossing. Then, each cock from A × B was mated with six hens from B × A and vice versa. [file 1471-2164-14-151-S4.pdf]

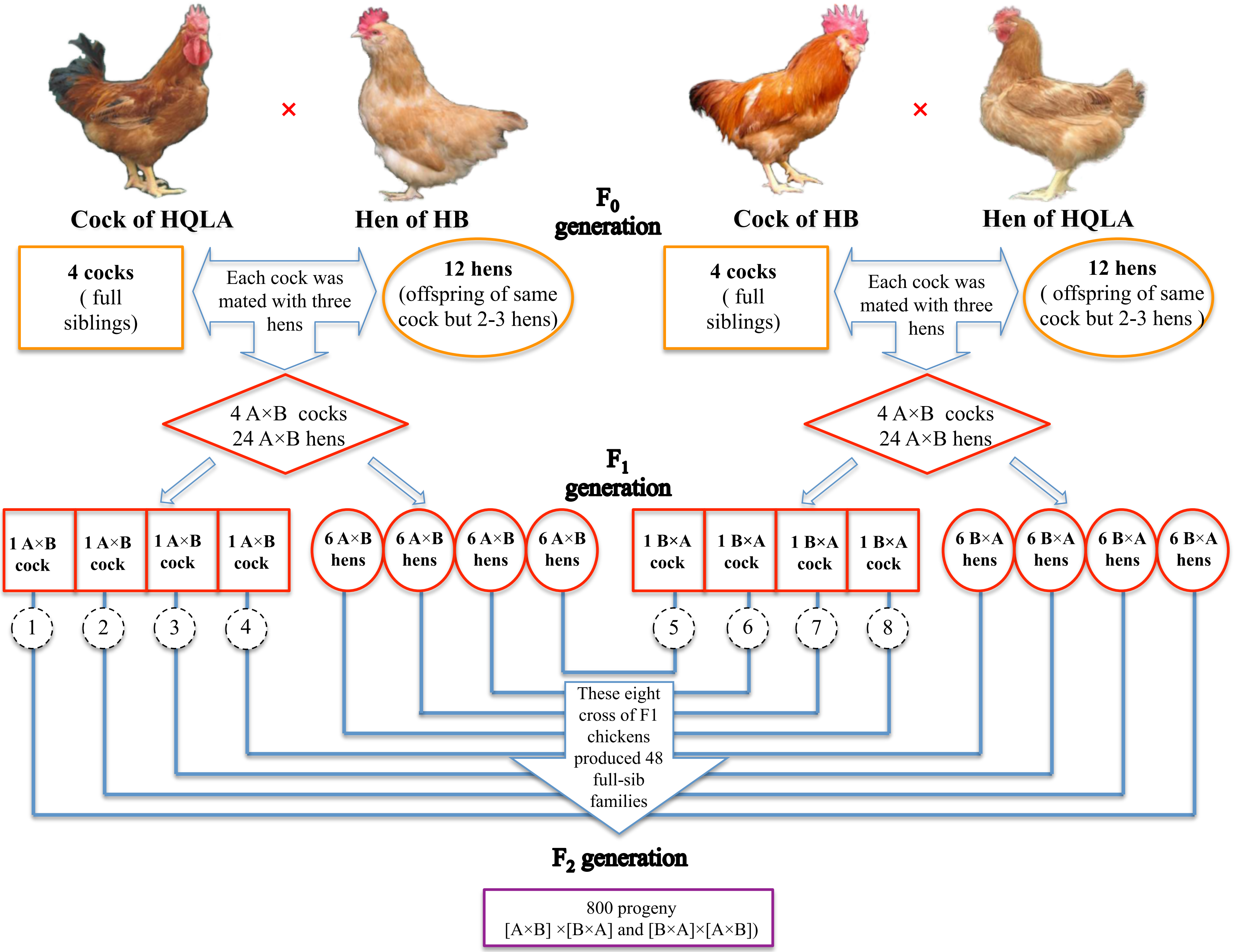
Cock of HQLA
Hen of HB
F0 generation
Cock of HB
Hen of HQLA
4 cocks (full siblings)
Each cock was mated with three hens
12 hens (offspring of same cock but 2-3 hens)
4 cocks (full siblings)
Each cock was mated with three hens
12 hens (offspring of same cock but 2-3 hens)
4 A×B cocks 24 A×B hens
F1 generation
4 A×B cocks 24 A×B hens
1 A×B cock
1 A×B cock
1 A×B cock
1 A×B cock
6 A×B hens
6 A×B hens
6 A×B hens
6 A×B hens
1 B×A cock
1 B×A cock
1 B×A cock
1 B×A cock
6 B×A hens
6 B×A hens
6 B×A hens
6 B×A hens
1
2
3
4
5
6
7
8
These eight cross of F1 chickens produced 48 full-sib families
F2 generation
800 progeny [A×B] ×[B×A] and [B×A]×[A×B])
